# Gender, work, and satisfaction: a decomposition approach to job satisfaction gaps in Egypt and Tunisia

**DOI:** 10.3389/fsoc.2025.1573489

**Published:** 2025-07-22

**Authors:** Mesbah Fathy Sharaf, Abdelhalem Mahmoud Shahen

**Affiliations:** ^1^Department of Economics, Faculty of Arts, University of Alberta, Edmonton, AB, Canada; ^2^Department of Economics, College of Business, Imam Mohammad Ibn Saud Islamic University (IMSIU), Riyadh, Saudi Arabia

**Keywords:** Egypt, Tunisia, job satisfaction, decomposition analysis, sample selection

## Abstract

**Introduction:**

This study revisits the paradox of the contented female worker by analyzing gender disparities in job satisfaction in Egypt and Tunisia.

**Methods:**

Using nationally representative labor force survey data, we construct a multidimensional job satisfaction index based on eight dimensions: earnings, job security, nature of work, working hours, work schedule, work environment, commuting distance, and job-qualification match. To explain gender gaps in job satisfaction, we apply the Blinder–Oaxaca decomposition method, both with and without correcting for sample selection bias.

**Results:**

Our results show that conclusions about the existence and direction of the gender gap depend critically on accounting for selection effects. Before correcting for selection bias, women in Egypt report significantly higher job satisfaction than men, while no gender gap is observed in Tunisia—echoing the contented female worker paradox. However, once sample selection is controlled for, the paradox disappears in both countries. In Egypt, the observed gender gap is fully explained by differences in observable characteristics (endowment effect), while in Tunisia, it is largely driven by differences in returns to those characteristics (coefficient effect), highlighting structural inequalities in the labor market.

**Discussion:**

To test the robustness of our results, we also conduct the decomposition using an alternative measure of job satisfaction based on a single overall satisfaction question. The consistency of results across both measures reinforces the validity of our conclusions. Together, these findings caution against relying solely on standard models of job satisfaction and emphasize the importance of considering sample selection and multidimensional outcomes. The study underscores the need for policy interventions that promote fairer working conditions, expand access to employment benefits, and address gender-based disparities in labor markets.

## 1 Introduction

Job satisfaction, broadly defined as the extent to which individuals find their work meaningful, rewarding, and fulfilling, plays a critical role in shaping labor market behaviors and outcomes. A substantial body of empirical literature has demonstrated strong links between job satisfaction and key labor outcomes such as productivity, turnover, absenteeism, and engagement in training and professional development (Judge et al., 2017; Bryson et al., 2017). Conversely, persistent dissatisfaction at work has been associated with negative consequences for employee wellbeing, including psychological stress, anxiety, and depression, as well as reduced firm performance (Ford et al., 2018; Silla et al., 2009; Faragher et al., 2005). As such, job satisfaction serves as both an individual-level welfare indicator and an important measure of organizational and economic health.

While gender disparities in labor market outcomes are well documented across a wide range of contexts—encompassing wage gaps, occupational segregation, and differential access to training and promotions (Blau and Kahn, 2017; Goldin, 2021)—less consistent attention has been paid to gender differences in subjective job satisfaction. This is particularly relevant in the case of Arab countries, where women face numerous structural barriers to employment and career advancement. In Egypt, for instance, female labor force participation remains among the lowest globally, at approximately 18%, with many women engaged in informal or precarious employment lacking access to legal protections and benefits (Zeitoun et al., 2023; Assaad and Krafft, 2013). Tunisia presents a somewhat different context, with higher female participation (around 25%) and greater access to public sector employment, yet gender gaps in earnings, leadership, and job security persist (OECD, 2022; Moghadam, 2021). These differing structural and policy contexts make Egypt and Tunisia compelling cases for comparative analysis.

The selection of these two countries also allows for an examination of gendered labor market experiences within similar cultural settings but under varying institutional conditions. Egypt and Tunisia share regional, religious, and historical commonalities, yet differ in terms of state capacity, gender policy, and economic reform trajectories. Compared to Western economies, where gender equality in the workplace is often supported by stronger legal enforcement and institutional protections, women in both Egypt and Tunisia continue to face deeply rooted societal and structural challenges. These contrasts highlight the importance of analyzing how gendered patterns of job satisfaction unfold in under-researched Arab labor markets.

A puzzling finding in the international literature is that, despite their relative disadvantages in the labor market, women often report similar or even higher levels of job satisfaction than men—a phenomenon widely referred to as the “gender–job satisfaction paradox” (Clark, 1997; Bender et al., 2005). Several explanations have been proposed, including lower job expectations among women, stronger valuation of non-monetary job attributes such as flexibility and stability, or differing reference groups and adaptation mechanisms (Mueller and Kim, 2008; Redmond and McGuinness, 2020; Dockery and Buchler, 2023). However, many of these studies do not adequately address the issue of sample selection bias—that is, the possibility that only a select group of women, who may have more favorable working conditions or intrinsic motivation, are represented in satisfaction data (Boll et al., 2017). Failing to account for this can result in misleading conclusions regarding true gender disparities in job satisfaction.

While this study corrects for sample selection bias using the Heckman two-step method, it is important to acknowledge that not all sources of bias may be fully addressed. Selection into employment among women may be influenced not only by job preferences—such as a desire for flexible hours or proximity to home—but also by broader structural and social constraints such as family responsibilities or limited access to suitable jobs that affect the probability of employment itself. Additionally, job satisfaction responses can be correlated with income, as individuals with higher earnings may be more likely to report being satisfied, independent of other job features. Although our model adjusts for observable selection based on employment status, these unobserved or partially measured factors may still influence the findings.

Understanding the gender–job satisfaction paradox is not merely of theoretical interest; it carries significant policy implications. If women appear more satisfied despite poorer job quality, this could mask structural inequalities and reduce the perceived urgency for labor market reforms. Moreover, interpreting satisfaction at face value may obscure the constraints and limited opportunities that shape women’s career paths. Addressing this paradox is therefore essential for developing more effective, equity-focused labor policies.

This study contributes to the literature in several key ways. First, while the paradox has been widely studied in developed and emerging economies, empirical research from the Arab region remains limited. By focusing on Egypt and Tunisia, this paper adds valuable comparative evidence from two distinct Arab labor markets. Second, we employ a methodological framework that corrects for sample selection bias using the Heckman two-step procedure (Heckman, 1979), thereby addressing a common limitation in the literature. Third, we apply the Blinder-Oaxaca decomposition technique (Blinder, 1973; Oaxaca, 1973) to distinguish between explained and unexplained components of the job satisfaction gap, allowing us to isolate the extent to which observed differences are due to individual and job characteristics versus potential structural or discriminatory factors.

Together, these contributions offer new insights into gender inequality in job satisfaction and help inform policies aimed at improving labor market conditions and gender equity in the Middle East and North Africa (MENA) region.

The remainder of this paper is organized as follows. Section 2 reviews the existing literature on gender disparities in job satisfaction. Section 3 outlines the data sources and methodology. Section 4 presents the empirical results. Section 5 discusses the findings in relation to existing research and policy implications. Section 6 concludes.

## 2 Literature review

Though there is a substantial literature on the determinants of job satisfaction in a wide range of countries (see, for example, Haile, 2015; Ezzat and Ehab, 2019), and for a recent systematic review of the empirical literature on the determinants of job satisfaction see Putra et al. (2023), empirical evidence on the gender–job satisfaction paradox is sparse in the case of Egypt and Tunisia.

Aside from studying the determinants of job satisfaction, a growing number of studies have emerged to examine the gender–job satisfaction paradox with inconclusive findings. While the paradox of the contented female worker was supported by several studies (see, for example, Bender et al., 2005; Carvajal et al., 2021;Yang et al., 2024), other studies find no statistically significant gender gap in the level of job satisfaction (see, for example, Unluoglu, 2022). A third group of studies found that the existence of a gender gap is country- and domain-specific, and is sensitive to the underlying methodology and the inclusion of job satisfaction determinants (see, for example, Akbari et al., 2020; Dilmaghani, 2022).

In an earlier study, Bender et al. (2005) found support for the paradox of the contented female worker in the U. S. They reported that women exhibit higher job satisfaction levels than men and that women working in female-dominated workplaces report even higher satisfaction. The study attributes this to job flexibility in such workplaces.

Using data from the 2014 European Skills and Jobs Survey covering 28 EU countries, Redmond and McGuinness (2020) also found that women, on average, report higher job satisfaction than men—even after controlling for personal, job-related, and family characteristics. However, this gender gap disappeared when job preferences were added, indicating that women place greater importance on work-life balance and intrinsic work rewards.

Asadullah and Talukder (2019), using employee-level data from 50 garment factories in Bangladesh, also supported the paradox. Female workers were found to be more satisfied with life and finances and less depressed than male workers. This gap remained robust after accounting for workplace policies such as childcare availability, female supervisors, and factory fixed effects.

Similarly, Carvajal et al. (2021) examined gender differences in career satisfaction among U. S. pharmacists, controlling for earnings, work hours, and other job-related characteristics. Their findings revealed that female pharmacists report greater satisfaction. Moreover, the study highlighted gender differences in responsiveness to workplace incentives—implying that a uniform incentive policy may not be equally effective for men and women.

Other studies found no statistically significant relationship between gender and job satisfaction. For instance, Unluoglu (2022) conducted a survey of employers in the tourism sector in Eskişehir (Turkey) and found no gender differences in satisfaction. Likewise, Boucher (2024) examined job satisfaction during the COVID-19 shift to remote work and found declines in job satisfaction for both genders, but no gender-based differences.

Dilmaghani (2022), using the 2016 Canadian General Social Survey, found a gender gap favoring women. However, when stratified by age and education, the gap disappeared. A Blinder-Oaxaca decomposition showed that intrinsic job rewards increased the explained portion of the gap.

The third strand of the literature emphasizes cross-country and domain-specific variation in the paradox. Kose and Avcioglu (2023), using data from 35 OECD countries, employed the Balanced Worth Vector method and found that the gender satisfaction gap differs not only across countries but also across job dimensions. In some countries, women report higher satisfaction, in others, men do, and in a third group, no significant difference is found. This mixed findings regarding the gender gap in job satisfaction implies that the effect of gender on job satisfaction is country specific and does not have a uniform pattern globally.

Akbari et al. (2020) analyzed domain-specific satisfaction among 146 nurses in Tehran. While overall satisfaction was lower among women, they reported higher satisfaction with certain dimensions—such as relationships and emotional aspects of nursing work.

Antón et al. (2023), using the European Working Conditions Survey (2005–2015), found that women enjoy better non-monetary working conditions, including physical environment and work-life balance, but face disadvantages in autonomy and career prospects.

Finally, Andrade et al. (2022), using ISSP data across 37 countries, reported that women in the hospitality industry had lower satisfaction than men in most occupations—except for receptionists, housekeeping supervisors, and cleaners.

After reviewing this literature, it is clear that a common shortcoming across studies is the neglect of sample selection bias—particularly the non-random selection of women into employment. Ignoring this bias may lead to inaccurate estimates of gender differences in job satisfaction. The current study aims to overcome this limitation by applying a Heckman-corrected decomposition model to control for such bias in the Egyptian and Tunisian contexts.

## 3 Materials and methods

### 3.1 Data sources

This study uses data from the 2012 round of the Egypt Labor Market Panel Survey (ELMPS) (OAMDI, 2024) and the 2014 round of the Tunisia Labor Market Panel Survey (TLMPS) (OAMDI, 2016). Both surveys are nationally representative and collect extensive information on individuals and households, with a strong focus on labor market characteristics. The surveys are conducted by the Economic Research Forum (ERF) in collaboration with national agencies and are available through the ERF Open Access Micro-Data Initiative. For detailed documentation, see Assaad and Krafft (2013) for ELMPS and Assaad et al. (2016) for TLMPS.

The job satisfaction questions are administered to all individuals who reported being employed during the 3 months preceding the interview. Our analytical sample consists of 9,988 wage employees in Egypt and 2,710 in Tunisia.

### 3.2 Measurement of job satisfaction

Job satisfaction is measured through both “overall” and “domain-specific” self-reported indicators, which have been shown to be valid and reliable (Spector, 1997). The overall job satisfaction measure captures general perceptions of one’s job, while the domain-specific indicators reflect satisfaction with specific aspects such as earnings, job security, and working conditions.

The main outcome variable is a job satisfaction index (JS), constructed by aggregating satisfaction scores across eight job-related domains: earnings, job security, type of work, number of working hours, work schedule, working conditions, commuting distance, and job-qualification match. Each domain is measured on a 5-point Likert scale ranging from “fully dissatisfied” (−2) to “fully satisfied” (+2). The overall index is created by summing the recoded values, resulting in a composite score that ranges from −16 to +16 as shown in Equation 1. This approach follows previous work by Bryson et al. (2013) and Haile et al. (2015). Table 1 lists all the job satisfaction-related questions.


(1) $${\mathrm{JS}}_{i,j}=\sum_{k=1}^{8}D_{i,j}^{k}$$

**Table 1:** Job satisfaction–related questions.

No.	Question
1	How satisfied are you with your current job?
2	How satisfied are you with job security?
3	How satisfied are you with earnings (wages)?
4	How satisfied are you with the type of work you do?
5	How satisfied are you with the number of working hours?
6	How satisfied are you with your work schedule?
7	How satisfied are you with working conditions/environment?
8	How satisfied are you with the distance to job/commuting?
9	How satisfied are you with the match between your qualifications and position?

Source: Egypt Labor Market Panel Survey (2012), Tunisia Labor Market Panel Survey (2014).

Where $D_{i,j}^{k}$ is the score for the answer to the domain-specific job satisfaction question by employee *i* in the domain k working in an occupation j.

### 3.3 Decomposition methodology

To explain the gender gap in job satisfaction, we apply the Blinder-Oaxaca decomposition method (Blinder, 1973; Oaxaca, 1973), a widely used econometric technique in labor economics. This method is particularly helpful in identifying the sources of differences in average outcomes—such as job satisfaction—between two groups. In our case, the groups are male and female workers.

The key insight behind this method is that any observed difference in average job satisfaction between men and women may arise from two broad sources: (a) differences in characteristics (known as the endowment or explained effect)—e.g., if men and women have different levels of education, working hours, or access to job benefits and (b) differences in how those characteristics translate into satisfaction (known as the coefficient or unexplained effect)—e.g., if women’s access to medical insurance affects their job satisfaction differently than it does for men, possibly due to workplace biases or structural discrimination.

This technique enables us to decompose the gender gap in job satisfaction into parts that are due to observable differences and those that may reflect deeper structural issues in the labor market.

Let job satisfaction JS be determined by a set of characteristics X, with group-specific coefficients $\beta^{\text{male}}$ for men and $\beta^{\text{female}}$ for women. We estimate separate regression equations for both groups as displayed in Equations 2, 3:


(2) $${\mathrm{JS}}^{\text{male}}=X^{\text{male}}\beta^{\text{male}}+\in^{\text{male}}$$

(3) $${\mathrm{JS}}^{\text{female}}=X^{\text{female}}\beta^{\text{female}}+\in^{\text{female}}$$

The average gender gap in job satisfaction is then defined as in Equation 4:


(4) $$\Delta ={\bar{\mathrm{JS}}}^{\text{male}}-{\bar{\mathrm{JS}}}^{\text{female}}$$

This observed gap Δ can be decomposed into three components as displayed in Equation 5.


(5) $$\Delta =({\bar{X}}^{\text{male}}-{\bar{X}}^{\text{female}})\;{\hat{\beta }}^{*}+{\bar{X}}^{\text{female}}({\hat{\beta }}^{\text{male}}-{\hat{\beta }}^{\text{female}})+R$$

where the first term $({\bar{X}}^{\text{male}}-{\bar{X}}^{\text{female}})\;{\hat{\beta }}^{*}$ is the explained or endowment effect: the part of the gap due to differences in average characteristics between men and women. The second term ${\bar{X}}^{\text{female}}({\hat{\beta }}^{\text{male}}-{\hat{\beta }}^{\text{female}})$ is the unexplained or coefficient effect: the part due to differences in how those characteristics affect job satisfaction. The third term R is the interaction term, which accounts for the fact that both characteristics and their returns can differ simultaneously.

We use the threefold decomposition framework, which separates all three effects—explained, unexplained, and interaction—to provide a detailed view of the sources of inequality. This decomposition approach provides a more accurate understanding of gender inequality in the labor market. Instead of just observing whether women are more or less satisfied with their jobs, we can ask: Is this because they have different types of jobs? Or because they are treated differently, even when they have similar jobs? This distinction is crucial for designing targeted policy interventions.

### 3.4 Correcting for sample selection bias

An important issue in studying job satisfaction is sample selection bias. In many settings—especially in the Middle East and North Africa—not all individuals, particularly women, participate in the labor market. Those who do may be a selective group, e.g., women who are willing to work only under favorable conditions. This means the sample of employed women may not be representative of all women, potentially biasing results.

To address potential sample selection bias—especially the non-random participation of women in the labor market—we employ the Heckman two-step correction model (Heckman, 1979) within the Blinder-Oaxaca decomposition framework. In the first step, a probit model estimates the probability of being employed based on a range of individual and job-related characteristics, including health status, education level, household wealth, workplace size, supervisory duties, sector of employment, commuting time, and access to benefits such as paid leave and medical insurance. These variables reflect factors that influence labor force participation decisions.

Specifically, the selection equation includes variables reflecting human capital and labor market access, such as self-reported health status (very good, good, fair), education levels (five categories from no diploma to university), household wealth score, and a range of workplace and job characteristics (e.g., rural job location, firm size, supervisory responsibilities, commuting time, night shifts, and sector of employment). Additionally, access to employment benefits such as paid sick leave, paid vacation, medical insurance, labor union membership, social insurance registration, formal appointment status, and temporary employment are included, alongside daily working hours. These variables are chosen to reflect the multifaceted decision to participate in the labor market, which is not random but shaped by both economic incentives and socio-cultural constraints, especially for women.

The selection equation is estimated using a probit model, and the inverse Mills ratio from this first stage is then included in the job satisfaction regression to correct for selection bias. This correction accounts for the fact that observed job satisfaction may not be representative of the entire population but only of those selected into employment.

In the second step, the job satisfaction equation is estimated, incorporating the inverse Mills ratio from the first stage to correct for selection bias. The model is implemented using the oaxaca command in Stata with the heckman, twostep option and the select subcommand specifying the probit model for employment.

While this approach helps correct for observable selection into employment, it is important to recognize that not all sources of bias may be fully addressed. For example, women’s employment decisions may also be shaped by preferences for flexible work or proximity to home, as well as structural constraints such as caregiving responsibilities or limited access to suitable jobs. Moreover, job satisfaction may be correlated with income, as higher earners might be more inclined to report being satisfied regardless of other job features. Although the model controls for observed selection, these unmeasured or partially observed factors may still influence the results and should be interpreted with appropriate caution.

## 4 Empirical results

### 4.1 Descriptive statistics

Table 2 presents descriptive statistics for key individual and workplace characteristics, disaggregated by gender and country. These variables provide important context for interpreting the gender gap in job satisfaction and understanding the role of selection bias.

**Table 2:** Descriptive statistics by gender and country.

Variable	Egypt—males	Egypt—females	Tunisia—males	Tunisia—females
Age (years)	36.96	38.80	40.15	37.24
Sex (% of total)	80.84	19.16	75.77	24.23
Rural residence (%)	58.18	50.15	32.23	26.90
Urban residence (%)	41.82	49.85	67.77	73.10
Married (%)	74.50	70.29	67.62	51.71
Single/Divorced/Widowed (%)	25.50	29.71	30.42	48.29
Health: very good/excellent (%)	30.42	25.81	35.28	40.10
Health: good (%)	53.37	54.93	51.05	43.27
Health: fair (%)	13.87	16.75	11.43	14.32
Health: bad/very bad (%)	2.35	2.51	2.24	2.31
Education: illiterate/no diploma (%)	25.21	28.19	13.04	17.24
Education: university and above (%)	17.35	32.32	5.93	14.74
Household wealth index	−0.026	0.253	0.04	0.09
Workplace: rural (%)	37.39	38.32	22.11	19.61
Workplace: urban (%)	48.22	59.72	70.70	69.03
Workplace: mobile (%)	14.39	1.96	7.19	11.36
Establishment size: 1–9 employees (%)	60.07	41.36	50.19	38.12
Establishment size: 10–99 employees (%)	16.65	28.80	14.78	20.45
Establishment size: 100 + employees (%)	9.34	11.80	14.54	23.16
Supervisory role (% yes)	19.12	14.18	16.39	12.41
Average working hours/day	8.64	4.29	3.76	3.67
Commute time (minutes)	31.12	23.18	19.44	18.98
Night work (% yes)	45.30	18.82	25.12	12.63
Sector: government (%)	27.36	70.45	16.78	21.47
Sector: public enterprise (%)	5.58	3.60	7.01	6.80
Sector: private (%)	64.47	23.32	44.32	38.98
Paid sick leave (% yes)	41.70	77.35	31.55	39.25
Paid vacation (% yes)	42.53	78.23	31.56	41.51
Medical insurance (% yes)	30.86	50.53	41.73	41.85
Union member (% yes)	16.77	27.62	5.27	6.87
Social insurance (% yes)	36.53	51.60	50.65	46.44
Formal contract (% yes)	43.44	81.42	34.82	47.12
Average daily wage	40.59	33.64	2.91	3.62

Source: authors’ calculation based on data from the 2012 round of ELMPS and the 2014 round of TLMPS.

The gender composition of the employed sample is heavily male-dominated in both countries, especially in Egypt, where only 19.2% of respondents are women. This reflects broader labor market realities and highlights the importance of correcting for selection bias, as employed women may represent a selective group.

Age differences are modest across groups, with Tunisian men slightly older on average. Residential patterns vary more significantly: while 58.2% of Egyptian men live in rural areas, 67.8% of Tunisian men live in urban areas. These patterns are mirrored among women and may influence job opportunities and satisfaction, as rural employment is often informal and lower in quality.

Marriage is more common among men in both countries, particularly in Tunisia, which may reflect cultural norms shaping labor force participation. Self-reported health status is generally positive, with most respondents rating their health as good or very good; Tunisian women report slightly better health than other groups, which is relevant as health is included in the selection equation of our Heckman model.

Educational attainment shows notable gender and regional variation. While many Tunisian men completed only elementary or middle school, a higher proportion of Egyptian women (32.3%) had university degrees compared to Egyptian men (17.4%). However, this advantage does not appear to translate into better job outcomes for women, pointing to potential mismatches between education and employment that may affect satisfaction.

Women in both countries tend to report higher household wealth scores, possibly reflecting selective labor force participation—where women from wealthier households may only work under favorable conditions.

Workplace characteristics also differ. Urban employment is more common in Tunisia. Although small firms dominate in both countries, large firms are more prevalent in Tunisia, especially for women. Nevertheless, women remain underrepresented in supervisory roles.

Working hours are significantly longer in Egypt (8.64 h per day for men) than in Tunisia (less than 4 h on average). Commute times are also longer in Egypt. Men are more likely to work night shifts, particularly in Egypt (45.3%).

Sectoral employment shows a sharp gender divide: Egyptian women are largerly concentrated in the public sector (70.5%), while men are more likely to work in the private sector. This may reflect both preferences for job stability and constraints on access to private employment.

Access to employment benefits—such as paid sick leave, paid vacation, and medical insurance—is generally higher among women, possibly because they are more often employed in formal sectors. However, union membership remains low overall, particularly in Tunisia.

Indicators of formal employment, such as social insurance and official contracts, are more common among women in both countries, supporting the idea that female employment is selective and more likely to occur in well-regulated segments of the labor market. Lastly, average daily wages are higher in Egypt than in Tunisia, and men earn more than women in both countries, reinforcing the income-based drivers of satisfaction gaps.

### 4.2 Job satisfaction patterns

**Figure 1:**
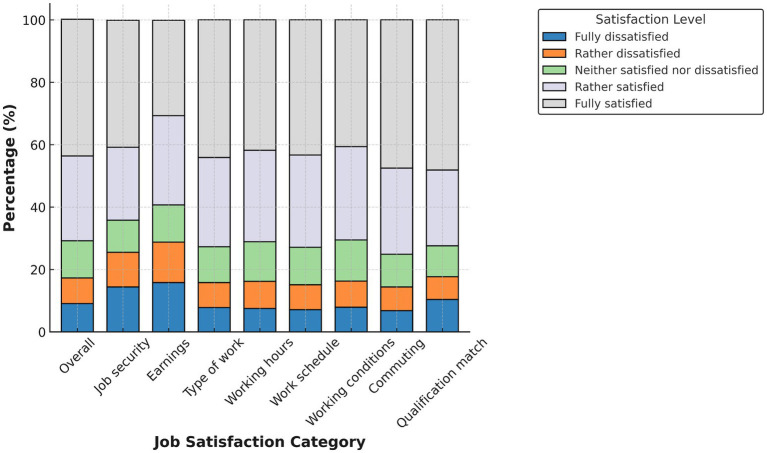
Job satisfaction in Egypt (males). Source: authors’ calculation based on data from the 2012 round of ELMPS.

**Figure 2:**
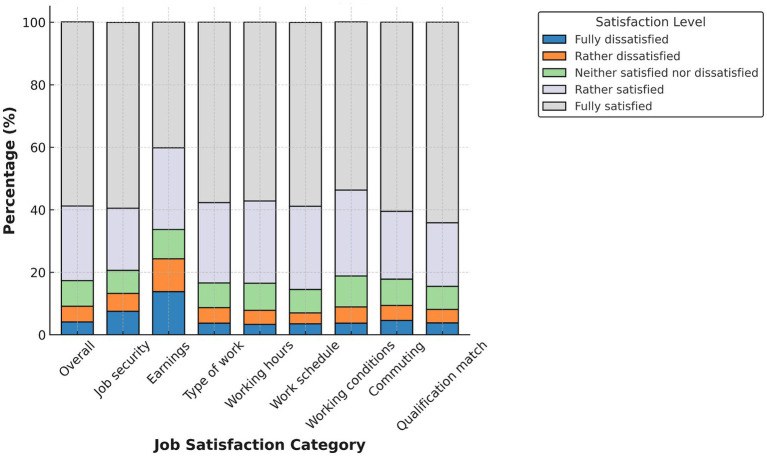
Job satisfaction in Egypt (females). Source: authors’ calculation based on data from the 2012 round of ELMPS.

**Figure 3:**
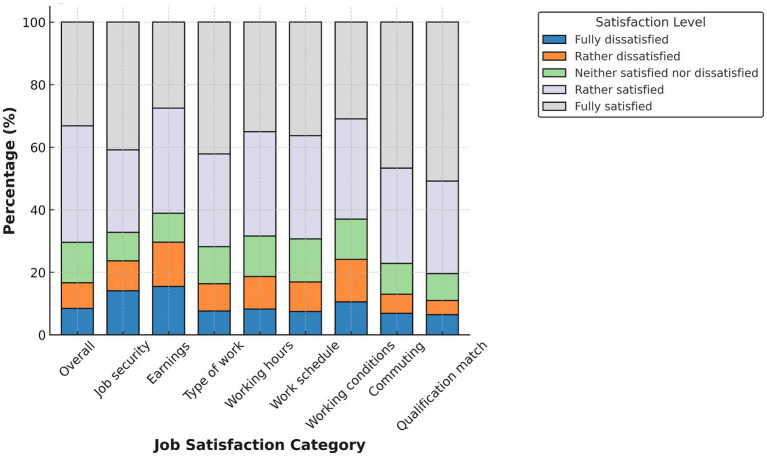
Job satisfaction in Tunisia (males). Source: authors’ calculation based on data from the 2014 round of TLMPS.

**Figure 4:**
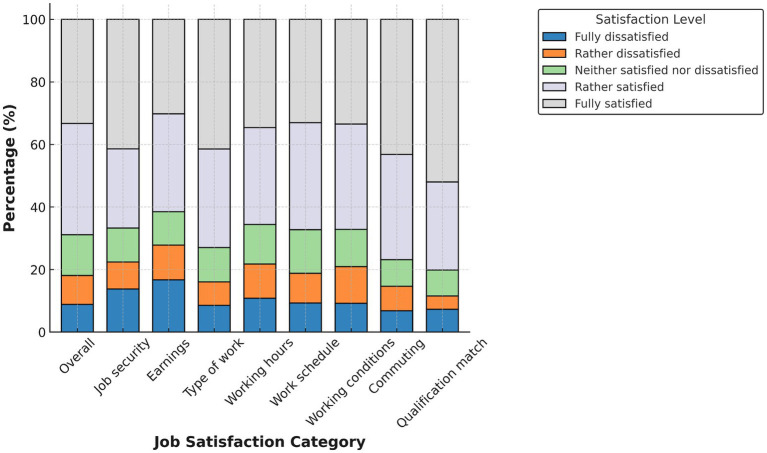
Job satisfaction in Tunisia (females). Source: authors’ calculation based on data from the 2014 round of TLMPS.

Figures 1–4 display domain-specific job satisfaction levels for men and women in Egypt and Tunisia. These descriptive patterns offer insights into how gendered experiences at work relate to overall satisfaction and align with the study’s main empirical findings.

In Egypt (Figures 1, 2), women report consistently higher satisfaction across nearly all job domains—including job security, working conditions, commuting time, and qualification match. More than half of female respondents report being “fully satisfied” in many of these areas. This high satisfaction among Egyptian women likely reflects their concentration in public sector jobs with more stability and benefits. In contrast, men report more dissatisfaction, particularly in earnings, working hours, and commuting—areas more associated with informal or demanding jobs.

These differences support the finding that the gender gap in Egypt is driven primarily by differences in job characteristics. Women, although fewer in number, are more likely to work in formal, stable positions that promote satisfaction.

In Tunisia (Figures 3, 4), gender differences are less pronounced and, in some domains, reversed. Men report higher satisfaction in areas such as qualification match, job security, and working conditions. A substantial share of women fall into the “rather dissatisfied” or “neutral” categories, especially regarding earnings, working hours, and the type of work. Despite having similar educational profiles, women appear to receive fewer returns in terms of satisfaction.

This supports our later decomposition results, which show that in Tunisia, the gender satisfaction gap is less about job characteristics and more about how men and women experience or are rewarded for similar attributes. This suggests the presence of structural discrimination or unmet expectations in the labor market.

### 4.3 Decomposition results

To quantify the sources of the gender gap in job satisfaction, we estimate Blinder-Oaxaca decompositions for both countries. We begin with the baseline model using OLS and then compare results with those from the Heckman two-step correction to account for selection bias.

As shown in Tables 3, 4, the OLS results reveal a statistically significant gender gap in Egypt of 1.76 points in favor of women. The majority of this gap is explained by endowments (1.80 points), with minimal contributions from coefficients or interaction effects. However, after applying the Heckman correction, the gap reverses to −8.14 points and becomes statistically insignificant. This reversal underscores the importance of accounting for selection into employment, especially given the highly selective nature of female employment in Egypt.

**Table 3:** Threefold decomposition of the gender gap in job satisfaction index in Egypt.

Component	OLS coefficient	OLS std. err.	Selection-corrected coefficient	Selection-corrected std. err.
Women	9.83***	0.17	−0.093	28.47
Men	8.06***	0.10	8.05***	0.10
Difference	1.76***	0.20	−8.14	28.47
Endowments	1.80***	0.15	1.98***	0.14
Coefficients	0.175	0.32	−11.81	34.48
Interaction	−0.216	0.29	1.68	6.01

Source: authors’ estimation. *** indicates significance at the 1% level.

**Table 4:** Threefold decomposition of the gender gap in job satisfaction index in Tunisia.

Component	OLS coefficient	OLS std. err.	Selection-corrected coefficient	Selection-corrected std. err.
Women	7.03***	0.47	3.97***	0.98
Men	7.81***	0.27	7.23***	0.21
Difference	−0.77	0.55	−3.26***	1.01
Endowments	−0.25	0.35	−0.55*	0.30
Coefficients	−0.111	0.62	−3.86***	1.45
Interaction	−0.42	0.55	1.16*	0.67

Source: authors’ estimation. ***, * indicate significance at the 1 and 10% level, respectively.

In Tunisia, the OLS model shows a smaller gap of −0.77 points against women. After selection correction, the gap widens to −3.26 and becomes statistically significant. Unlike Egypt, the unexplained component (coefficients effect) dominates the gap in Tunisia, indicating that women receive lower returns to similar job characteristics—potentially reflecting workplace discrimination or unequal treatment.

### 4.4 Robustness checks

To verify the robustness of our findings, we repeat the decomposition using an alternative outcome variable: the overall job satisfaction question. Results are reported in Tables 5, 6.

**Table 5:** Threefold decomposition of the gender gap in overall job satisfaction in Egypt.

Component	OLS coefficient	OLS std. err.	Selection-corrected coefficient	Selection-corrected std. err.
Women	4.31***	0.02	5.49	4.72
Men	4.10***	0.01	4.09***	0.01
Difference	0.20***	0.03	1.39	4.72
Endowments	0.14***	0.02	0.21***	0.02
Coefficients	0.03	0.06	1.46	5.98
Interaction	0.03	0.05	−0.27	1.26

Source: authors’ estimation. *** indicates significance at the 1% level.

**Table 6:** Threefold decomposition of the gender gap in overall job satisfaction in Tunisia.

Component	OLS coefficient	OLS std. err.	Selection-corrected coefficient	Selection-corrected std. err.
Women	3.92***	0.05	3.57***	0.15
Men	4.02***	0.03	3.95***	0.02
Difference	−0.09	0.06	−0.37***	0.15
Endowments	−0.06	0.03	−0.05	0.04
Coefficients	0.00	0.07	−0.47*	0.28
Interaction	−0.03	0.05	0.16	0.15

Source: authors’ estimation. ***, * indicate significance at the 1 and 10% level, respectively.

In Egypt, the OLS estimate of the overall satisfaction gap is 0.20 points in favor of women, with the gap again driven by differences in observable characteristics. As in the index-based model, the gap becomes statistically insignificant once selection is accounted for.

In Tunisia, the overall satisfaction model shows a statistically significant gender gap of −0.37 points after selection correction, consistent with the findings from the index-based model. Again, the coefficients component accounts for most of the explained variation.

These robustness checks confirm the reliability of our core findings: in Egypt, the observed gender gap is largely due to women working in better jobs, while in Tunisia, the gap reflects unequal treatment or valuation of similar characteristics.

## 5 Discussion

The findings of this study provide important insights into gender disparities in job satisfaction in Egypt and Tunisia. The results indicate that, prior to correcting for selection bias, women in Egypt appear to report higher levels of job satisfaction compared to men, while there is no statistically significant gender gap in Tunisia. This initially supports the well-documented paradox of the contented female worker, which suggests that women report higher job satisfaction despite facing worse labor market conditions (Clark, 1997; Bender et al., 2005). However, once selection bias is accounted for, the paradox no longer holds in either country. These results highlight the importance of considering sample selection effects when analyzing gender disparities in job satisfaction (Kaiser, 2007; Sousa-Poza and Sousa-Poza, 2003).

The decomposition analysis reveals key differences between the two countries. In Egypt, the gender gap in job satisfaction is fully explained by the endowment effect, suggesting that disparities arise from differences in job-related characteristics rather than differences in how men and women perceive their working conditions. This aligns with prior research indicating that women in Egypt are often concentrated in lower-quality jobs with fewer benefits and protections (Assaad et al., 2016; El-Hamidi, 2008). Conversely, in Tunisia, the coefficient effect plays a more significant role, suggesting that gender differences in job satisfaction persist due to structural discrimination and unequal treatment in the workplace, rather than differences in job characteristics. These findings are consistent with previous studies highlighting gender-based inequalities in job rewards and career advancement opportunities in Tunisia (Moghadam, 2021; Ferrer-i-Carbonell and Mora, 2009).

Moreover, dissatisfaction with earnings is the most prominent driver of job dissatisfaction in both Egypt and Tunisia. This is consistent with international labor market studies indicating that wage disparities significantly influence job satisfaction (Blau and Kahn, 2017; Goldin, 2021). However, other aspects, such as job security and working conditions, also contribute substantially to dissatisfaction, reinforcing concerns about labor market instability in both countries (Hersch and Xiao, 2016). The high rates of informal employment and lack of legal job protections, particularly for women, exacerbate these concerns. Addressing these disparities requires comprehensive labor policies that promote formal employment opportunities and provide better social protection for workers (Asadullah and Talukder, 2019).

Another important aspect of the findings is the role of workplace benefits. The study highlights that access to benefits such as medical insurance, paid leave, and social security plays a crucial role in job satisfaction. In both Egypt and Tunisia, women generally have less access to these benefits compared to men, contributing to their overall dissatisfaction (Antón et al., 2023). Policies aimed at reducing these inequalities, such as enforcing equal access to employment benefits and expanding coverage of labor laws to include informal workers, could improve job satisfaction for women and reduce the gender gap in employment conditions.

Finally, structural barriers and cultural expectations regarding gender roles in the labor market also contribute to gender differences in job satisfaction. In Egypt and Tunisia, social norms often influence women’s employment choices and job experiences, leading to different expectations and evaluations of job quality compared to men (Luo, 2016). While some women may report higher satisfaction despite worse working conditions due to lower expectations, correcting for selection bias in our study reveals the true extent of the gender gap. This underscores the need for policy interventions that address not only economic factors but also social and cultural dimensions of gender inequality in the workplace.

To ensure the robustness of these findings, we also compared the decomposition results using two distinct measures of job satisfaction: the multidimensional job satisfaction index (our main specification) and the overall job satisfaction question. The results from both measures are remarkably consistent. In Egypt, the gender gap remains positive and is largely explained by endowments under both specifications, while the selection-corrected model shows the gap becomes statistically insignificant in both cases. In Tunisia, both measures reveal a negative gender gap, and in both cases the coefficient effect is the primary contributor to the explained variation, especially after controlling for selection bias. These results validate our main conclusions and demonstrate that the findings are not driven by the particular choice of job satisfaction measure. Instead, they reflect underlying structural dynamics in gendered labor market experiences in both countries.

## 6 Conclusion

The paradox of the contented female worker—the idea that women report higher job satisfaction despite worse working conditions—has long puzzled researchers. This study re-examines that paradox in the contexts of Egypt and Tunisia, using nationally representative data and addressing a key methodological issue in prior research: the failure to account for sample selection bias. Our results show that ignoring selection bias leads to misleading conclusions. Once this bias is corrected, the paradox no longer holds. Men report higher levels of job satisfaction than women in both countries.

In Egypt, the gender gap in job satisfaction is primarily explained by differences in job characteristics. This finding suggests that women are more likely to occupy lower-quality jobs, such as those with fewer benefits, limited job security, and poorer working environments. In Tunisia, the gap is instead driven by differences in how job characteristics translate into satisfaction for men and women, indicating that structural discrimination and unequal treatment play a greater role.

These differences between countries point to the need for country-specific policy responses. In Egypt, efforts should focus on raising the overall quality of women’s employment. This involves expanding access to formal jobs, ensuring legal protections such as equal pay and social insurance, and improving work conditions in female-dominated sectors that are often undervalued. In Tunisia, where the unexplained gap reflects potential workplace discrimination, there is a stronger need for stricter enforcement of anti-discrimination laws, gender sensitivity training, and improved oversight of workplace practices.

At a broader level, both countries would benefit from policies that support women’s ability to participate fully and fairly in the labor market. For example, expanding access to childcare and promoting flexible work arrangements can help reduce the burden of unpaid care work, which remains a major barrier for many women. Encouraging the advancement of women into leadership roles can also help reduce gender gaps in both opportunity and satisfaction. These goals can be supported through targeted leadership development programs, gender quotas, and employer partnerships that invest in female talent across sectors.

While this study offers valuable insights into the drivers of gender disparities in job satisfaction, it is not without limitations. The use of cross-sectional data prevents us from observing how satisfaction and gender gaps evolve over time. Future research could benefit from longitudinal data that track workers across different stages of their careers. Qualitative research could also complement these findings by shedding light on how workplace culture, norms, and informal practices shape gendered experiences of satisfaction. Another important limitation of this study is the gender composition of the sample. In Egypt, 81% of the respondents are male and only 19% are female, while in Tunisia, 76% are male and only 24% are female. This reflects broader labor market patterns in both countries, where women have much lower labor force participation rates. However, such imbalances may limit the representativeness of the findings, especially when making gender comparisons. Although we address this issue methodologically through selection bias correction, the underrepresentation of women remains a constraint that future research should address. Studies using more balanced samples or oversampling of female respondents could provide further insight into the gender dynamics of job satisfaction.

In addition, although the Heckman model addresses bias from labor force selection, other sources of selection may remain. For example, women’s preference for flexible jobs or the tendency of higher earners to report greater satisfaction could influence the observed patterns. These unmeasured factors may introduce residual bias and should be explored further in future research, possibly through the use of panel data or qualitative methods.

In conclusion, our results show that looking only at what people say about their job satisfaction can hide deeper problems and unfair treatment in the labor market. A more careful and methodologically sound approach—such as the one taken in this study—is needed to reveal the real sources of gender gaps. Addressing these gaps requires both better job opportunities for women and stronger protections against discrimination, along with broader institutional reforms to promote fairness and equality in the world of work.

## Data Availability

Publicly available datasets were analyzed in this study. This data can be found at: https://erf.org.eg/labor-market-panel-surveys-lmps/.
